# Cortical auditory evoked potential in cochlear implant users: An objective method to improve speech perception

**DOI:** 10.1371/journal.pone.0274643

**Published:** 2022-10-07

**Authors:** Dayse Távora-Vieira, Andre Wedekind, Ellen Ffoulkes, Marcus Voola, Roberta Marino

**Affiliations:** 1 Fiona Stanley Fremantle Hospitals Group, Perth, Western Australia, Australia; 2 Division of Surgery, Medical School, The University of Western Australia, Perth, Australia; 3 Faculty of Health Sciences, School of Occupational Therapy, Social Work and Speech Pathology, Curtin University, Perth, Australia; Universidad de Chile, CHILE

## Abstract

**Objective:**

To investigate if cortical auditory evoked potential (CAEP) measures can be used to verify the cochlear implant (CI) map and consequently improve CI outcomes in adults with bilateral hearing loss.

**Design:**

CAEPs were measured in CI recipients using the speech tokens /m/, /g/, /t/ and /s/. If CAEP responses were present for all speech tokens, the participant’s map was considered “satisfactory”. If CAEP responses were absent, the CI map was considered “unsatisfactory” and therefore adjusted and CAEP measures repeated. This was repeated until auditory potentials were seen in response to all four speech tokens. Speech testing was conducted pre-CI, as well as before and after CAEP-guided map adjustments.

**Results:**

108 adult unilateral CI users participated, whose sound processors were previously programmed using subjective methods. 42 CI users elicited a CAEP response to all four speech tokens and therefore no further mapping adjustments were made. 66 subjected lacked a CAEP response to at least one speech token and had their CI map adjusted accordingly. Of those, 31 showed a CAEP response to all four speech tokens, and the average speech score significantly improved after CI map adjustments based on CAEP responses.

**Conclusion:**

CAEP’s are an objective tool that can be used to guide and verify CI mapping in adults CI users. Significant improvement in speech scores was observed in participants who had their CI map adjusted based on CAEP responses.

## Introduction

Cochlear implants (CI) are devices that bypass the inner ear and directly stimulate the auditory nerve. The electrical stimulation induces a pattern of activity that differs from acoustic stimulation but still mimics the tonotopic principles of the cochlea, allowing users to differentiate speech sound and to interpret auditory input [1]. CI is currently the most effective method of rehabilitation for hearing impaired individuals where amplification no longer provides meaningful benefit. Initially, CI candidacy included only those with severe to profound hearing loss. However, clinical research has led to a change in this perspective, broadening candidacy to include individuals with more residual hearing and focusing on speech understanding rather than the audiogram [2].

CI programming in adults relies heavily on subjective input provided by the patient. For instance, a CI is programmed through measuring the lowest current stimulation level that a patient can hear consistently (T levels) and the maximum comfortable level (C or MCL). MCLs are set with a minimal amount of current stimulation and gradually increased until the patient reports the sound is “loud but comfortable”, thereby creating an electrical dynamic range (DR) for each CI electrode [3]. However, this programming method is not standardized, with techniques varying widely both between individual clinicians and between CI clinics [4,5]. Vaerenberg et al., 2014 demonstrated that 31% of clinics around the world measured Ts only, and that 24% of clinics measured MCLs only, while 45% of clinics measured both Ts and MCLs. The study included clinics from Australia, Belgium, Canada, France, Germany, India, Italy, Lebanon, Morocco, Norway, Poland, Romania, Spain, The Netherlands, Turkey, United Kingdom, and USA.

It is also more challenging to elicit reliable subjective responses from paediatric patients or individuals with cognitive impairment [6], patients with tinnitus [7] or those with pre-lingual deafness [8]. Pierzycki et al., 2019 reported that 80% of audiologists and 45% of patients found that the presence of tinnitus made it more problematic to measure Ts, and that 26% of patients with tinnitus set their C/MCLs more conservatively, as they were concerned that louder stimulation would increase their tinnitus [7]. Duration of deafness can also be a factor, with Polak et al., 2006 demonstrating that prelingually deafened adults are more likely to have a smaller dynamic range than post lingually deafened patients, and also demonstrate greater variability for Ts and C/MCLs measurement.

It is possible that subjective CI programming can create a suboptimal mapping which favours comfort over speech perception, which can lead to a delay in language development in children [9]. CI programming based on objective measures may increase the reliability of responses, while also saving clinicians’ time [10].

Objective measures currently used to establish the T and C/MCL during CI map fitting include eSRT (electrically evoked stapedius reflex threshold), electrically evoked compound action potentials (eCAP) and electrically evoked auditory brainstem responses (eABRs). eSRT has been shown to correlate well with subjectively set MCLs in both paediatric and adult CI users [8,11–13]. However, it may be absent in as many as 30% of CI users, limiting its use for CI fitting and verification [14–18]. eCAPs have also been studied as an objective measure to find the electrical DR in CI users. However, eCAP thresholds correlate weakly with subjective Ts and MCLs [6,11] and are not deemed as good predictors of speech discrimination [19]. Similarly, eABRs have also been investigated as an objective tool for CI map fitting and verification, however they also correlate poorly with Ts and C/MCLs [20–22].

Numerous studies have investigated the use of Cortical Auditory Evoked Potentials (CAEPs), particularly the P1-N1-P2 complex, to verify speech detection in children and in adults receiving amplification [23–29].

The presence of CAEPs in response to a speech signal may indicate that speech is audible to the individual [27,30]. Oviatt & Kileny (1991) showed that CI users with good speech understanding exhibited similar amplitudes and latencies of the N1-P2 complex compared to normal hearing individuals. Likewise, Rance, Cone-Wesson, Wunderlich & Dowell (2002) demonstrated a clear relationship between speech perception scores and the presence of CAEPs. Further studies have investigated the potential benefits of using the P1-N1-P2 complex as an objective tool to verify CI performance [31]. CAEP Ts have been found to strongly correlate to behavioural Ts [19,32] giving significance to the presence/absence of the response during CI fitting or map verification.

In the present work, we sought to ascertain whether CAEPs can be used to verify the CI map and consequently improve CI users’ performance. This may help to mitigate the potential over- or under-stimulation of the auditory cortex that could occur with a map based on a subjective loudness perception scale.

## Methods

### Ethics

Ethics approval was obtained from the South Metropolitan Health Services Human Research Ethics Committee (reference number: 3258). Participants have given their written informed consent to participate in this study.

### Subjects

A total of 108 adult (68 males; 40 females) unilateral CI users with bilateral deafness were recruited from the audiology department at a tertiary teaching hospital. The demographic and clinical characteristics of the participants are summarized in Table 1. Mean age at testing was 65.9 ± 16.1 years. Mean duration of deafness was 23.2 ± 18.5 years (range 0.5–72 years) and CI experience 7 ± 2.9 years (range 0.4–16 years).

**Table 1:** Participant demographics and clinical characteristics.

Gender		Implanted Ear	
*Male *	68	*Right *	62
*Female *	40	*Left *	46
**Pure Tone Average (dB HL) **		**Aetiology**	
*Implanted Ear *	92.5 ± 18.9	*Autoimmune Disease *	6
*Contralateral Ear *	70.4 ± 27.9	*Congenital*	9
* *		*ISSNHL*	27
**Type of Implant **		*Meniere’s Disease *	12
*CONCERTO *	19	*Noise Induced Hearing Loss *	9
*SONATA *	2	*Presbycusis *	12
*SONATAti100 *	2	*Middle Ear Infection*	18
*SYNCHRONY*	85	*Other*	15

ISSNHL = idiopathic sudden sensorineural hearing loss. Aetiology of ‘Other’ includes meningitis, vascular, stroke and neuropathy.

All participants were implanted with MED-EL electrode arrays (MED-EL, Innsbruck, Austria). All participants had their CI programmed using the subjective method of measuring Ts and C/MCLs prior to taking part in this study.

Review of data logging and/or self-report of usage indicated that they used their speech processor during all waking hours.

Institutional ethics approval was obtained (reference number 3258). Written informed consent was obtained from all subjects.

### Cortical auditory evoked potentials

**Fig 1:**
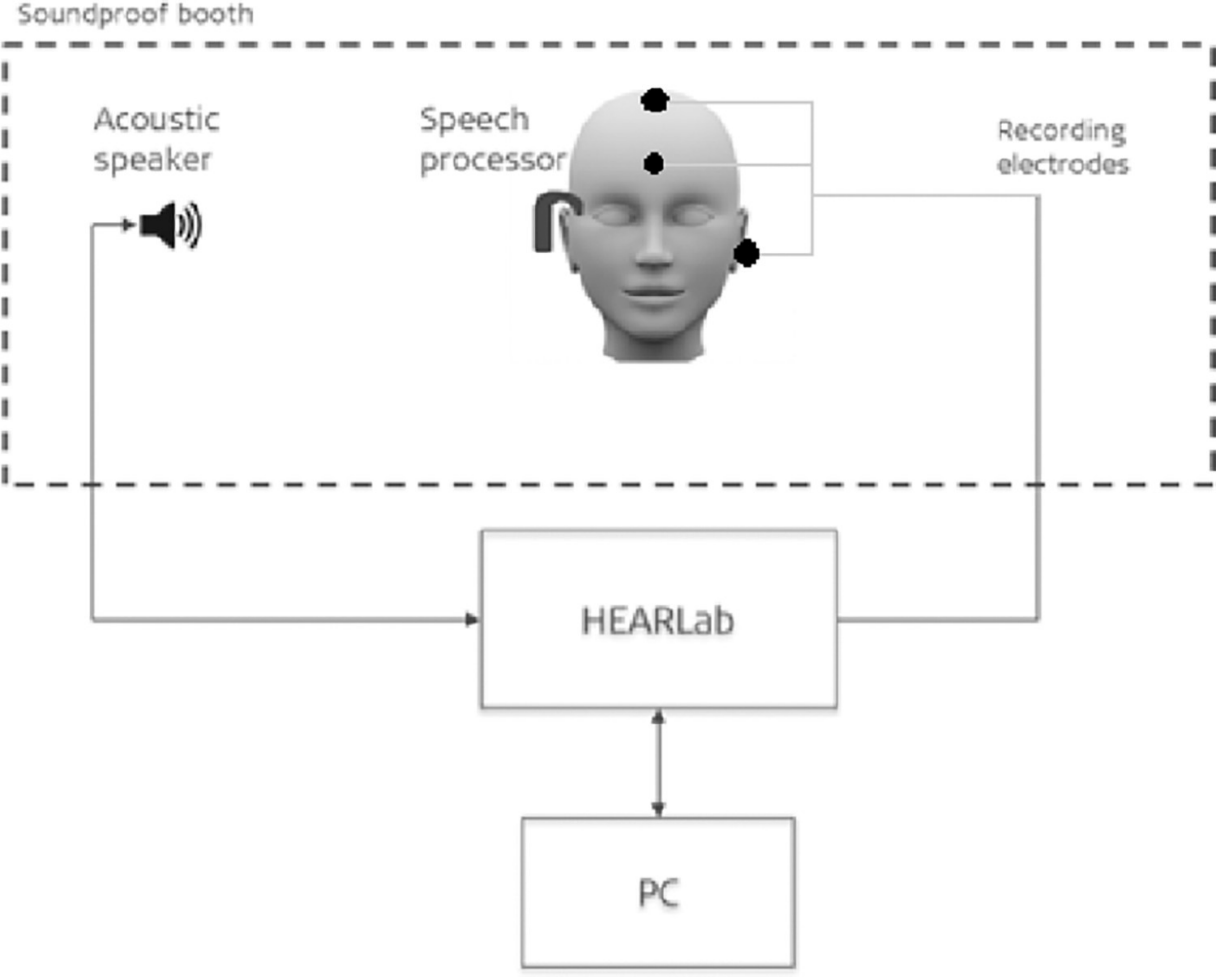
Electrode montage for CAEP recording. Active electrode at the vertex (Cz), reference on the mastoid contralateral to the CI and the ground placed at the forehead. Electroencephalogram (EEG) electrodes were used for the experiment.

Subjects were awake during the procedure. CAEPs were recorded using the HEARLab™ System (Frye Electronics, Tigard, Oregon) which applies an automatic statistical analysis to determine the presence or absence of CAEPs. It applies an automatic statistical analysis to determine the presence or absence of CAEPs. HEARLab relies on the Hotellings’s T^2^ test [33] which calculates the probability that the mean value of any linear combination of the bin variables is significantly different from noise, with a p-value < 0.05 indicating a significant result. The HEARLab System has been used to record CAEPs generated by acoustic stimulation [12,34] and can be used for testing normal hearing individuals or hearing aid or CI users. CAEPs can be elicited by presenting speech tokens with differing characteristic frequencies and pre-determined stimulation levels. This token presentation can be used to objectively evaluate if the stimulus activates the auditory cortex. Carter et al. (2013) demonstrated that the automated statistical detection of cortical responses used in the HEARLab system is as accurate as visual detection by three expert CAEP examiners [30]. Electrode impedance was kept below 5kΩ and the residual noise level below 3.2 μV. The number of accepted epochs in a test run was pre-set to 200 epochs per speech token. Recording electrodes placement is shown in Fig 1.

Testing was performed in free field using the HEARLab in-built speech tokens /m/, /g/, /t/ and /s/ presented at the soft level of 55 dB SPL with the speaker located at 0° azimuth. The frequency ranges of the tokens were /m/: 200–500 Hz, /g/: 800–1600 Hz, /s/: 2000–8000 Hz and /t/: 3000–8000 Hz. This was verified by FFT spectrum analysis.

Data was exported in.txt format for further analysis using Python 3.5 and the average for participants’ CAEP recording was obtained.

### Speech perception testing

The Consonant-nucleus-consonant (CNC) word test [35] was used to measure the ability to hear speech in quiet. A single speaker setup in free field was used with the speaker placed 1 metre away at 0° azimuth. Speech perception scores were obtained immediately before CAEP measurement and acutely after adjusting the CI map and CAEP recorded for all speech tokens. Different CNC word list was used for Pre- and Post-CAEP to account for any learning effects. Pre-Op results were obtained from medical records.

### Test procedure

**Fig 2:**
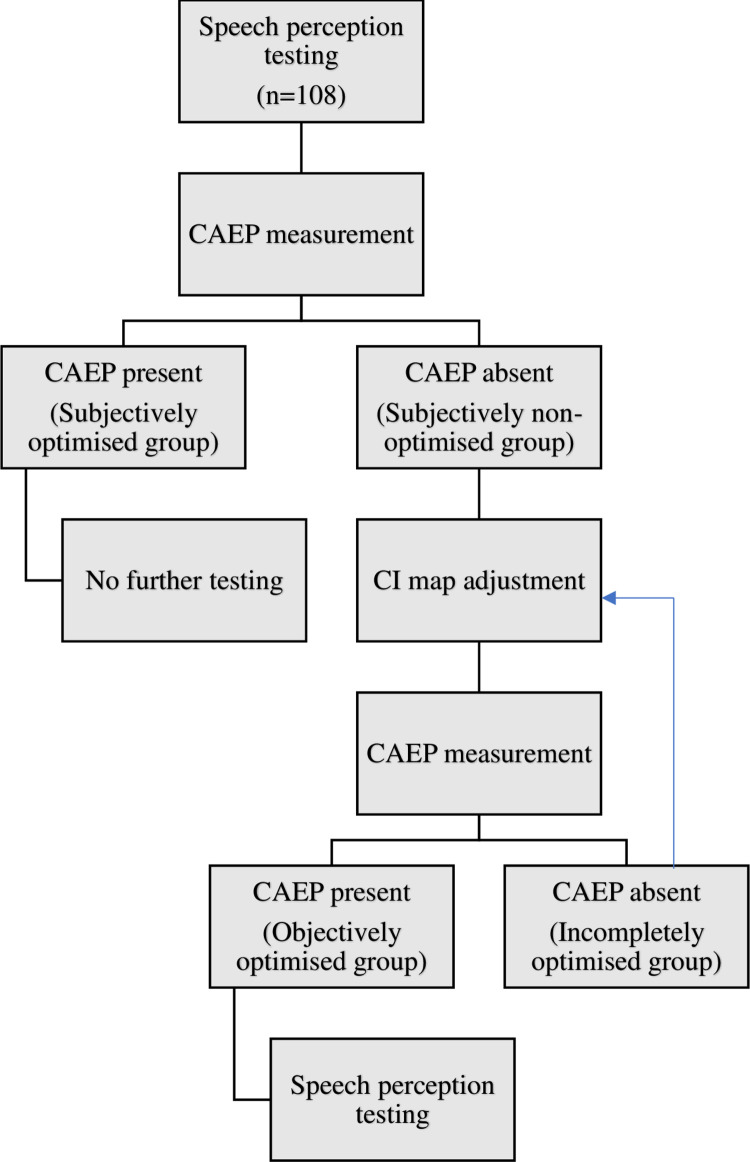
Visual representation of the testing protocol and the participants breakdown forming the four groups.

The test procedure is summarized in Fig 2. An implant audiologist programmed the implant and performed the CAEP recordings. To evaluate subjective based mapping, speech scores were tested for each participant prior to the CAEP guided mapping session. The CI user then underwent CAEP measurement for each speech token. If the HEARLab software indicated a cortical response (*p*-value < 0.05), visual inspection of the P1-N1-P2 complex was performed by two experienced audiologists, with one audiologist being blinded to the programming of the implant and to the HEARLab results.

The CI map was considered “satisfactory” if CAEP responses were computationally detected (*p*-value < 0.05) for all four speech tokens, and the waveform was considered to be a cortical response by at least one audiologist. In this case, no further adjustments or speech testing were performed. This sub-group of participants are referred to as *subjectively optimised*.

If, however, no CAEP responses were detected for one or more speech tokens and/or the two reviewing audiologists were unsure about the waveform, the CI map was modified by adjusting the MCLs for the cochlear implant electrode contacts corresponding to the frequency of the speech token(s) that did not evoke a cortical response. This sub-group is referred to as *subjectively non-optimised group*.

Subjects in *subjectively non-optimised group* were instructed to alert the audiologist if the new levels became uncomfortable or provoked non-auditory stimulation. CAEP measurement was performed after each adjustment, and subsequent adjustments were made until a CAEP was evoked or the patient rejected more changes due to discomfort or non-auditory perception. If CAEPs were recorded for all four speech tokens after MCLs adjustment, these participants were classified as *objective optimised group* and their post-CAEP speech perception scores were obtained. If CAEPs were still not recorded for one or more speech tokens, the participants were classified as *incompletely optimised group* and not further testing was performed.

### Statistics

Statistical analyses were conducted using R statistics and R Studio software [36]. Results were analysed using the ‘anova’ function and follow-up pairwise comparisons were conducted using the emmeans fucntion from the ‘emmenas’ package [37]. This function was used to examine the difference between group speech perception scores as well as comparison of pre-CAEP to post-CAEP speech perception scores within groups 3. A p- value of < 0.05 was considered statistically significant. The speech perception scores are shown as the mean ± standard deviation.

## Results

### CAEP by HEARLab

**Fig 3:**
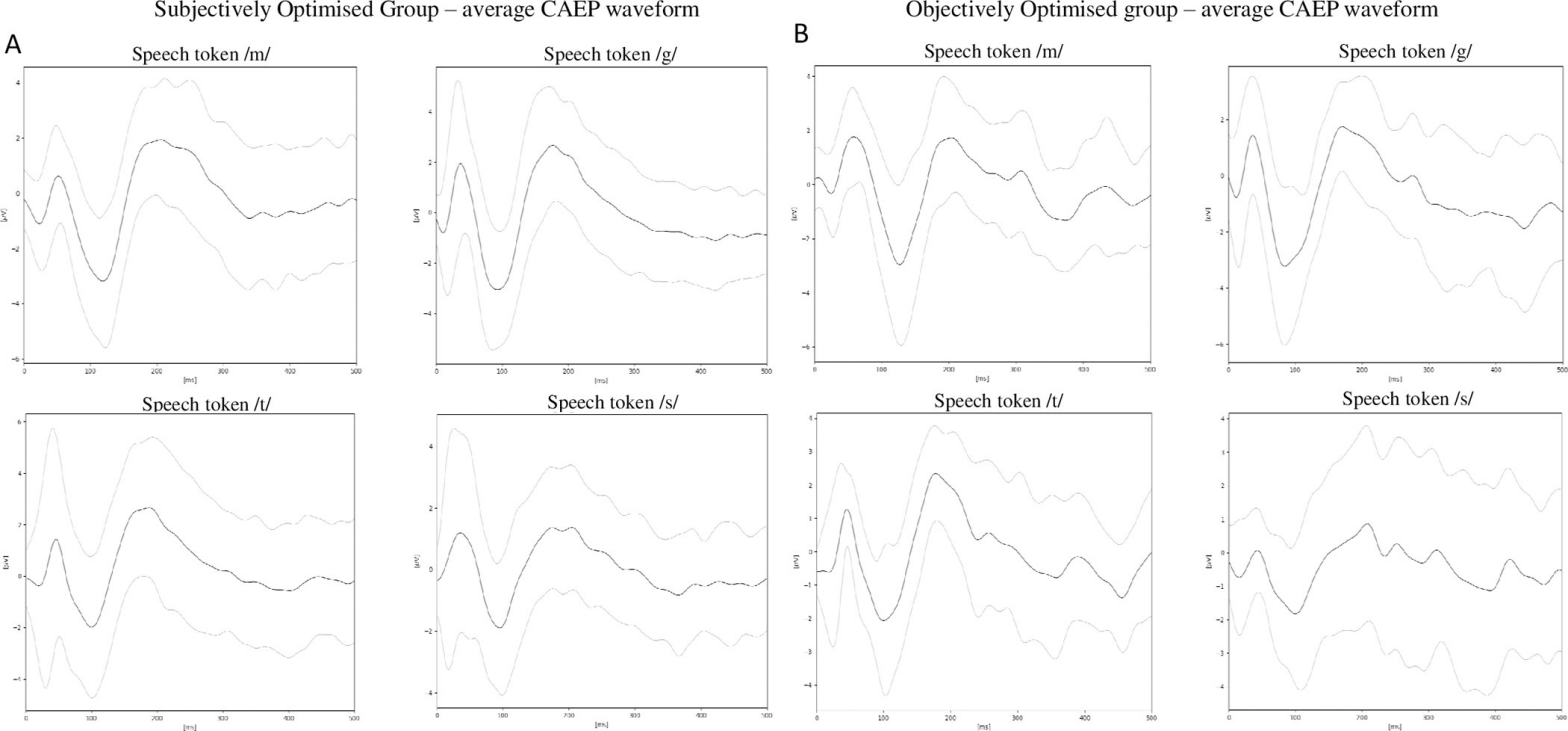
Average waveforms for the four speech tokens for the subjectively optimised and objectively optimised groups. The black line represents the group average waveform. The light grey lines represent group mean ± standard deviation.

CAEPs were recorded for all four speech tokens in 42 (39%) participants and therefore map adjustments were not required (*subjectively optimised*). CAEPs were not recorded for at least one speech token in the remaining 66 (61%) participants and therefore had their CI map adjusted (*subjectively non-optimised group*). Of the 66, 31 (47%) participants obtained CAEP responses from each speech token after adjustments to their map (*objective optimised group*). In the remaining 35 participants (53%), CAEP responses could not be elicited for at least one speech token despite CI map adjustment (*incompletely optimised group*). This is detailed in Table 2. The number of participants who had absent CAEP response for one, two, three or four speech tokens in each group are shown in Table 3. The mean and standard deviation of the CAEP waveform for all participants who had CAEPs elicited by all four speech tokens (*subjectively optimised* and *objectively optimised*) are shown in Fig 3.

**Table 2:** Distribution of participants who had absent CAEP for each speech token.

Groups	Participants (n)	Participants with CAEP response absent (n)
Subjectively optimised	42	0
Subjectively non-optimised	66	/m/ - 25 /g/ - 26 /t/ - 36 /s/ - 57
Objectively optimised	31	0
Incompletely optimised	35	/m/ - 5 /g/ - 6 /t/ - 11 /s/ - 30

**Table 3:** Distribution of participants who had CAEP response absent for one, two, three or four speech tokens.

Tokens missing	Subjectively optimised	Subjectively non-optimised	Objectively optimised	Incompletely optimised
1 token	0	24	0	25
2 tokens	0	19	0	7
3 tokens	0	11	0	0
4 tokens	0	12	0	3

**Fig 4:**
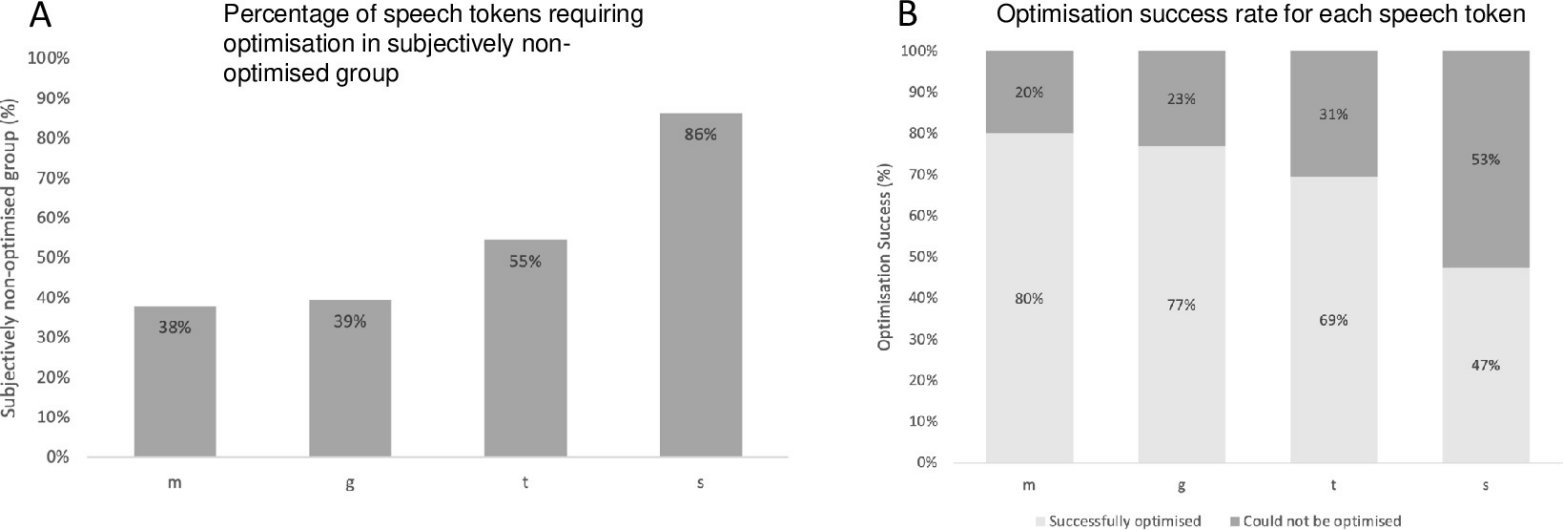
(A) Percentage of CAEP responses for the four speech tokens obtained from the subjectively non-optimised group. (B) Percentage of CAEPs obtained for each speech token post-CAEP guided mapping.

Within the group of 66 participants who did not have CAEP responses elicited by at least one speech token (*subjectively non-optimised group*), they were most often absent for /s/ (86% of the participants) followed by /t/ (55%), /g/ (39%) and /m/ (38%) (Fig 4A and Table 2). After CI adjustments, CAEP responses remained absent for /s/ in 53% of the participants, for /t/ in 31%, for /g/ in 23% and for /m/ in 20%. A summary of which speech tokens could elicit a CAEP responses are shown in Fig 4B.

Details of the number of participants who did not show CAEP response for a certain number of tokens in each group are depicted in Table 3.

### Speech outcome improvement

**Fig 5:**
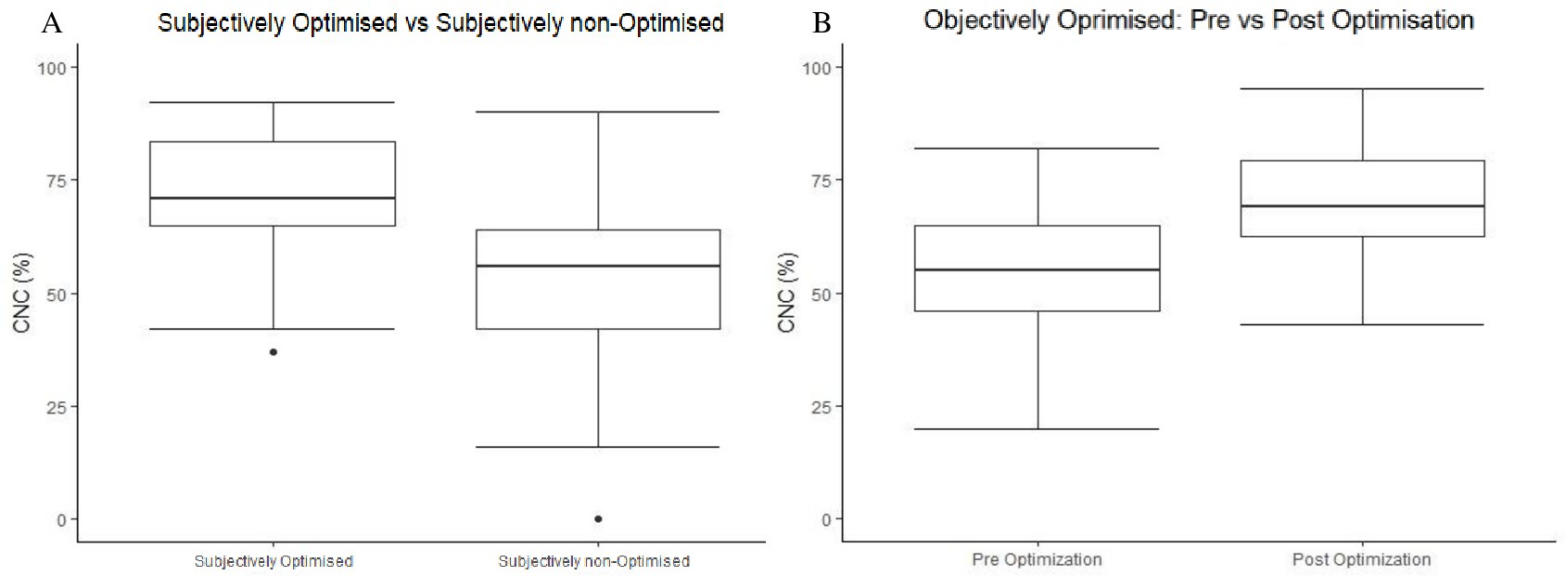
(A) Difference in speech perception scores between the subjectively optimised group and the subjectively non-optimised group. (B) Difference in speech perception scored between the objective optimised group pre-CAEP to post-CAEP.

Speech perception scores were obtained for all participants prior to CAEP measurement. The group average scores for the entire group improved from 14% ± 18 pre-CI to 63% ± 21 post-CI (t(70) = 11.2, *p < 0*.001)). The average for the *subjectively optimised group* significantly improved from 10% ± 15 pre-CI to 70% ± 15 post-CI (t(29) = 19, *p < 0*.001)); and for the *subjectively non-optimised group* significantly improved from 17% ± 21 pre-CI to 50% ± 19 post-CI (t(31) = 6.84, *p < 0*.*001*). A comparison between the *subjectively optimised group* and the *subjectively non-optimised group* showed that the *subjectively optimised group* achieved a higher group average speech perception score than *the subjectively non-optimised group* which was statistically significant (t(44.6) = 17.3, *p* < 0.001) shown in Fig 5A.

Withing the *subjectively non-optimised group*, those who had CAEP responses elicited by all speech tokens following the map adjustment (*objective optimised group*) had an improvement in speech perception scores from 19% ± 19 pre-CI to 54% ± 17 post-CI (t(20) = 9.6, *p* < 0.001). After CAEP measurements and map adjustments there was a further increase from 54% ± 17 pre-CAEP to 70% ± 13 post-CAEP which was statistically significant (t(23) = -5.668, *p* < 0.001) shown in Fig 5B. Only one participant in the *objective optimised group* did not show any improvement in speech perception scores from pre-CAEP to post-CAEP.

Those who continued having a CAEP response absent for at least one speech token (*incompletely optimised group*) had an improvement in speech perception scores from 17% ± 20.6 pre-CI to 49% ± 17.4 post-CI (t(26) = 4.454, *p* < 0.001). No speech test was performed after incomplete optimisation.

There was an improvement in speech scores regardless of duration of CI experience. CI users with less than 12 months experience showed a significant improvement in speech scores (t(24) = 2.480, *p* < 0.021) and CI users with greater than 12 months experience also experienced a significant improvement in speech scores (t(20) = 2.767, *p* < 0.012). There was no significant difference in the average improvement in speech scores between the two groups (t(22) = 0.594, *p* < 0.559).

## Discussion

Several studies have investigated the use of CAEPs, particularly the P1-N1-P2 complex, to verify speech detection in children and in adults receiving amplification [23,24,26,28,29,38,39]. In line with this, this study investigated the use of CAEPs to verify CI fitting and consequently improve hearing outcomes in a wide population of adult CI users.

CAEPs responses were present for all four speech tokens in 42 (39%) participants, suggesting that their CI map, which was based on subjective loudness perception, was capable of eliciting CAEPs responses and no map adjustments were needed. In contrast, 66 of the participants (61%) required CI map adjustments before CAEPs responses could be elicited. This suggests that the behavioural CI programming method was not effective for a large proportion of participants and might contribute to the variability of CI outcomes. From these 66 individuals almost half (n = 31) had CAEP responses elicited by the four speech tokens after CI map adjustments. These findings suggest that a CI map could be more accurate when using objective measurement as previously suggested [40].

It has been previously established that the presence of a CAEP response reflects the arrival and perception of sound at the higher levels of the brain [31,41]. If a CAEP response is elicited by a speech signal, it means that speech is audible and available for processing at a cortical level [30,39]. Considering this, when a speech token did not elicit a CAEP response it is possible that the CI user was unable to detect sound at the frequency range specific to that speech token. To verify this, our study looked at the speech perception scores of our entire group as well as each subgroup.

Regardless of CAEP measurement, the entire cohort achieved a significant improvement in speech scores from pre-CI to post-CI. When divided into two sub-groups, both *subjectively optimised group and subjectively non-optimised group* showed a significant improvement from pre-CI to post-CI speech scores. However, the *subjectively non-optimised group* had an average speech perception score substantially lower compared to the *subjectively optimised group*. It is well established that several factors can affect CI outcomes. However, absent CAEPs could indicate that the CI map was not providing enough stimulation to the higher orders of the brain involved in CAEP responses to speech sound, resulting in a “unsatisfactory” electrical stimulation and consequently compromised CI outcomes.

When CI map adjustments resulted in CAEP responses being present (*objective optimised group*), a significant improvement in speech scores from before to after adjustments was found. This finding provides valuable evidence that the use of objective measures in CI programming, specifically CAEPs, may be used to verify CI fitting and provide improved CI performance. Furthermore, mapping adjustments made to elicit CAEP responses to all four speech tokens in the *objective optimised group* resulted in an average speech score very similar to those in the *subjectively optimised group*. This further highlights the advantage of verifying CI map using an objective tool. It is important to note that all participants were seen regularly for CI programming based on loudness perception methods prior to being enrolled in this study. Therefore, it is unlikely that the same improvement could be achieved using subjective methods.

It is worth noting that in this study only those who had CAEPs elicited by all four speech tokens were included in the *objective optimised group*. As demonstrated in Table 3, the number of speech tokens that did not elicit a CAEP response decreased from the *subjectively non-optimised group* to the *incompletely optimised group*. It is reasonable to expect that improvement would be seen in speech perception scores. Nevertheless, this study did not investigate that as no speech perception scores were obtained for the *incompletely optimised group*. Therefore, further studies would benefit from investigating whether the presence of P1-N1-P2 complex for less speech tokens (*incompletely optimised group*) would improve speech perception scores.

Although investigation whether CI experience would affect the CAEP recording in adults was not part of the initial plan for this study, it was thought that CI users with long CI experience could potentially find difficult to adapt to CI map adjustments based on presence/absence of CAEP responses. The participants in this study had different CI experience. However, due to the limited number of participants within the 6–12 months bracket, we divided the group into >12 months and <12 months of CI experience, with 61 participants having less than 12 months, and 47 having more than 12 months of CI use. Our results demonstrated an improvement in speech scores regardless of duration of CI experience. There was no significant difference in the average improvement in speech scores between the two groups. This raises an important question regarding when a CI user’s improvement plateaus. While it has generally been considered that long term CI users reach a plateau in their speech discrimination scores, our results would suggest that in some cases there is room for further improvement regardless of user experience and time post-implantation.

Analysis of which speech token was more or less likely to elicit a CAEP response demonstrated that /m/ and /g/ were the most common ones followed by /t/, with CAEP elicited by /s/ remaining absent for more than 50% of those who had their CI map adjusted. It is also observed that the poorest CAEP morphology was obtained for /s/. Although direct comparison cannot be made due to different population (adult vs children), these findings differ from those reported by Kosaner et al., 2018 who showed that CAEP responses were mostly elicited by /t/ followed by /g/ and /m/ (/s/ was not tested in their study). Kosaner used an objective method (eSRT) to create the CI map and then CAEP to verify the audibility, therefore there was no subjective behavioural method involved contrary to our study [42].

Anecdotally, in our clinical experience, it is a common observation that CI users tend to have low tolerance to high frequency electrical stimulation and might request lower C/MCLs during behavioural map adjustments. It is also possible that within our adult population several had progressive hearing loss with the high frequencies being the first affected and therefore longer deprivation to high frequency sounds, making the adaptation to these sounds longer. It is also possible that CAEPs are affected by the bandwidth and duration of the stimulus. According to the HEARLab manual, /s/ has the stimulus duration of 50ms, /g/ 20 ms, /t/ 30 ms, and /m/ 30 ms. As reported by Tavora-Vieira et al. (2021), the artifact of the HEARLab recordings starts from 0 ms onwards, and it is larger for the speech token /s/, probably due to the longer stimulus duration [43]. Nevertheless, clinicians might consider working with these CI users to gradually improve their acceptance to high frequencies sounds to see if a CAEP response can be elicited. In other words, it is important to consider not only comfort but also audibility with CAEPs being a useful tool to guide this output.

In our study none of the participants who had their map fully adjusted based on the CAEP measures have requested to return to the previous subjectively set CI map. Many patients reported they were pleased to see that there was an objective method to check if the brain was detecting the sound and were more accepting of programming changes, particularly when their comfort levels were being increased. This was also true for the *incompletely optimised group*. Although no speech scores post-CAEP were obtained for the *incompletely optimised group*, it is reasonable to infer that they found some subjective benefits from the partial optimisation as they all accepted the CI map modifications.

One of the main barries for using CAEP responses to verify CI mapping is the additional time taken for the procedure, which varied from 15 to 45 minutes based on how many times the MCLs had to be adjusted before CAEPs responses were obtained. While this was a time-intensive process, the time savings attainable in the future could be considerable. At CI activation, many patients are given successively louder maps to adjust to. Using a CAEP guided map as the foundation for these initially fitted maps could significantly decrease programming time required in subsequent sessions. This is particularly useful for patients living in remote locations and for those not willing or able to attend regular programming and rehabilitation sessions.

This study has provided evidence that CAEPs can be utilised to verify CI fitting and improve CI users’ speech perception scores. However, an important limitation of this study is that only speech perception in quiet scores were evaluated, while speech perception in noise would provide a better indication of how the CI users are performing in real life situation. Further research is necessary to investigate the most adequate time during the rehabilitation program post-CI that CAEP can be used, whether it is useful during first fitting and factors influencing the presence/absence of CAEPs, and the cost-effectiveness of early optimisation. A randomised study with a group receiving the conventional subjective mapping method and another group receiving objective mapping method and evaluating the effect P1-N1-P2 amplitude on CI outcome would be of great value for future research.

## Conclusion

CI use provides a significant improvement on speech perception scores for bilateral deaf recipients. However, speech outcomes may be further enhanced by ensuring that each patient’s CI map is delivering a “satisfactory” auditory signal to the auditory cortex. CAEPs are a useful objective tool to verify and improve CI fitting.

## Supporting information

S1 Table Raw data on participants demographics and the speech token present (1) or absent (0) before and after CAEP measurement and CI optimisation. (XLSX)

S2 Table Means and standard deviation for the speech perception scores for the subjectively optimised group, subjectively non-optimised group and before/after optimisation for the objectively optimised group. (XLSX)

## References

[1] Kral Sharma. Developmental neuroplasticity after cochlear implantation.(Report). Trends in Neurosciences. 2012;35(2):111. doi: 10.1016/j.tins.2011.09.004 22104561 PMC3561718

[2] Vickers D, De Raeve L, Graham J. International survey of cochlear implant candidacy. Cochlear Implants Int. 2016;17 Suppl 1:36–41. Epub 2016/04/22. doi: 10.1080/14670100.2016.1155809 . 27099109

[3] Van Eeckhoutte M, Spirrov D, Wouters J, Francart T. Objective binaural loudness balancing based on 40-Hz auditory steady-state responses. Part II: Asymmetric and bimodal hearing. Trends in Hearing. 2018;22:2331216518805363. doi: 10.1177/2331216518805363 30334496 PMC6196612

[4] Vaerenberg B, Smits C, De Ceulaer G, Zir E, Harman S, Jaspers N, et al. Cochlear implant programming: a global survey on the state of the art. ScientificWorldJournal. 2014;2014:501738. Epub 2014/04/02. doi: 10.1155/2014/501738 ; PubMed Central PMCID: PMC3932199. 24688394 PMC3932199

[5] Browning LM, Nie Y, Rout A, Heiner M. Audiologists’ preferences in programming cochlear implants: A preliminary report. Cochlear Implants Int. 2020;21(4):179–91. Epub 2020/01/05. doi: 10.1080/14670100.2019.1708553 . 31900086

[6] de Vos JJ, Biesheuvel JD, Briaire JJ, Boot PS, van Gendt MJ, Dekkers OM, et al. Use of electrically evoked compound action potentials for cochlear implant fitting: a systematic review. Ear and hearing. 2018;39(3):401–11. doi: 10.1097/AUD.0000000000000495 28945656

[7] Pierzycki RH, Corner C, Fielden CA, Kitterick PT. Effects of Tinnitus on Cochlear Implant Programming. Trends Hear. 2019;23:2331216519836624. Epub 2019/03/19. doi: 10.1177/2331216519836624 ; PubMed Central PMCID: PMC6423681. 30880643 PMC6423681

[8] Polak M, Hodges AV, King JE, Payne SL, Balkany TJ. Objective methods in postlingually and prelingually deafened adults for programming cochlear implants: ESR and NRT. Cochlear implants international. 2006;7(3):125–41. doi: 10.1179/cim.2006.7.3.125 18792380

[9] Caner G, Olgun L, Gültekin G, Balaban M. Optimizing fitting in children using objective measures such as neural response imaging and electrically evoked stapedius reflex threshold. Otol Neurotol. 2007;28(5):637–40. Epub 2007/08/02. doi: 10.1097/mao.0b013e3180577919 . 17667772

[10] Presacco A, Innes-Brown H, Goupell MJ, Anderson S. Effects of stimulus duration on event-related potentials recorded from cochlear-implant users. Ear and hearing. 2017;38(6):e389. doi: 10.1097/AUD.0000000000000444 28475545 PMC5659925

[11] Polak M, Hodges A, Balkany T. ECAP, ESR and subjective levels for two different nucleus 24 electrode arrays. Otology & Neurotology. 2005;26(4):639–45. doi: 10.1097/01.mao.0000178145.14010.25 16015160

[12] Kosaner J, Van Dun B, Yigit O, Gultekin M, Bayguzina S. Clinically recorded cortical auditory evoked potentials from paediatric cochlear implant users fitted with electrically elicited stapedius reflex thresholds. International journal of pediatric otorhinolaryngology. 2018;108:100–12. Epub 2018/04/02. doi: 10.1016/j.ijporl.2018.02.033 . 29605337

[13] Walkowiak A, Lorens A, Polak M, Kostek B, Skarzynski H, Szkielkowska A, et al. Evoked stapedius reflex and compound action potential thresholds versus most comfortable loudness level: assessment of their relation for charge-based fitting strategies in implant users. ORL. 2011;73(4):189–95. doi: 10.1159/000326892 21659787

[14] Battmer R-D, Laszig R, Lehnhardt E. Electrically elicited stapedius reflex in cochlear implant patients. Ear Hear. 1990;11(5):370–4. doi: 10.1097/00003446-199010000-00008 2262087

[15] Bresnihan M, Norman G, Scott F, Viani L. Measurement of comfort levels by means of electrical stapedial reflex in children. Archives of Otolaryngology–Head & Neck Surgery. 2001;127(8):963–6. doi: 10.1001/archotol.127.8.963 11493206

[16] Hodges AV, Balkany TJ, Ruth RA, Lambert PR, Dolan-Ash S, Schloffman JJ. Electrical middle ear muscle reflex: use in cochlear implant programming. Otolaryngology-Head and Neck Surgery. 1997;117(3):255–61. doi: 10.1016/s0194-5998(97)70183-9 9334774

[17] Spivak LG, Chute PM. The relationship between electrical acoustic reflex thresholds and behavioral comfort levels in children and adult cochlear implant patients. Ear Hear. 1994;15(2):184–92. doi: 10.1097/00003446-199404000-00008 8020651

[18] Caner G, Olgun L, Gültekin G, Balaban M. Optimizing fitting in children using objective measures such as neural response imaging and electrically evoked stapedius reflex threshold. Otol Neurotol. 2007;28(5):637–40. doi: 10.1097/mao.0b013e3180577919 17667772

[19] Abbas PJ, Brown CJ. Assessment of responses to cochlear implant stimulation at different levels of the auditory pathway. Hear Res. 2015;322:67–76. doi: 10.1016/j.heares.2014.10.011 . 25445817 PMC4380632

[20] Brown CJ, Hughes ML, Lopez SM, Abbas PJ. Relationship between EABR thresholds and levels used to program the CLARION speech processor. Ann Otol Rhinol Laryngol Suppl. 1999;177:50–7. Epub 1999/04/24. doi: 10.1177/00034894991080s411 . 10214802

[21] Gordon KA, Papsin BC, Harrison RV. Toward a battery of behavioral and objective measures to achieve optimal cochlear implant stimulation levels in children. Ear Hear. 2004;25(5):447–63. Epub 2004/12/16. doi: 10.1097/01.aud.0000146178.84065.b3 . 15599192

[22] Lundin K, Stillesjö F, Rask-Andersen H. Prognostic value of electrically evoked auditory brainstem responses in cochlear implantation. Cochlear Implants Int. 2015;16(5):254–61. Epub 2015/03/24. doi: 10.1179/1754762815Y.0000000005 . 25798647

[23] Purdy SC, Kelly AS, Thorne PR. Auditory evoked potentials as measures of plasticity in humans. Audiol Neurootol. 2001;6(4):211–5. Epub 2001/11/06. doi: 10.1159/000046835 . 11694730

[24] Sharma A, Dorman MF, Kral A. The influence of a sensitive period on central auditory development in children with unilateral and bilateral cochlear implants. Hear Res. 2005;203(1–2):134–43. Epub 2005/04/28. doi: 10.1016/j.heares.2004.12.010 . 15855038

[25] Golding M, Doyle K, Sindhusake D, Mitchell P, Newall P, Hartley D. Tympanometric and acoustic stapedius reflex measures in older adults: the Blue Mountains Hearing Study. J Am Acad Audiol. 2007;18(5):391–403. Epub 2007/08/25. doi: 10.3766/jaaa.18.5.4 . 17715649

[26] Glista D, Easwar V, Purcell DW, Scollie S. A Pilot Study on Cortical Auditory Evoked Potentials in Children: Aided CAEPs Reflect Improved High-Frequency Audibility with Frequency Compression Hearing Aid Technology. Int J Otolaryngol. 2012;2012:982894. Epub 2012/12/01. doi: 10.1155/2012/982894 ; PubMed Central PMCID: PMC3501956. 23197983 PMC3501956

[27] Korczak PA, Kurtzberg D, Stapells DR. Effects of sensorineural hearing loss and personal hearing AIDS on cortical event-related potential and behavioral measures of speech-sound processing. Ear Hear. 2005;26(2):165–85. Epub 2005/04/06. doi: 10.1097/00003446-200504000-00005 . 15809543

[28] Alvarenga KF, Amorim RB, Agostinho-Pesse RS, Costa OA, Nascimento LT, Bevilacqua MC. Speech perception and cortical auditory evoked potentials in cochlear implant users with auditory neuropathy spectrum disorders. Int J Pediatr Otorhinolaryngol. 2012;76(9):1332–8. doi: 10.1016/j.ijporl.2012.06.001 . 22796193

[29] Chang HW, Dillon H, Carter L, van Dun B, Young ST. The relationship between cortical auditory evoked potential (CAEP) detection and estimated audibility in infants with sensorineural hearing loss. Int J Audiol. 2012;51(9):663–70. Epub 2012/08/10. doi: 10.3109/14992027.2012.690076 . 22873205

[30] Carter L, Dillon H, Seymour J, Seeto M, Van Dun B. Cortical auditory-evoked potentials (CAEPs) in adults in response to filtered speech stimuli. Journal of the American Academy of Audiology. 2013;24(9):807–22. doi: 10.3766/jaaa.24.9.5 24224988

[31] Kelly AS, Purdy SC, Thorne PR. Electrophysiological and speech perception measures of auditory processing in experienced adult cochlear implant users. Clinical Neurophysiology. 2005;116(6):1235–46. doi: 10.1016/j.clinph.2005.02.011 15978485

[32] Visram AS, Innes-Brown H, El-Deredy W, McKay CM. Cortical auditory evoked potentials as an objective measure of behavioral thresholds in cochlear implant users. Hear Res. 2015;327:35–42. doi: 10.1016/j.heares.2015.04.012 . 25959269

[33] Golding M, Pearce W, Seymour J, Cooper A, Ching T, Dillon H. The relationship between obligatory cortical auditory evoked potentials (CAEPs) and functional measures in young infants. J Am Acad Audiol. 2007;18(2):117–25. doi: 10.3766/jaaa.18.2.4 . 17402298

[34] Távora-Vieira, Wedekind A, Marino R, Purdy SC, Rajan GP. Using aided cortical assessment as an objective tool to evaluate cochlear implant fitting in users with single-sided deafness. PLoS ONE. 2018;13(2):e0193081–e. doi: 10.1371/journal.pone.0193081 . 29470548 PMC5823436

[35] Peterson GE, Lehiste I. Revised CNC lists for auditory tests. J Speech Hear Disord. 1962;27(1):62–70. doi: 10.1044/jshd.2701.62 14485785

[36] R TR. A language and environment for statistical computing. Vienna, Austria: 2013.

[37] Lenth R, Singman H, Love J, Buerknerm P, Herve M. emmeans: Estimated Marginal Means, aka Least-Squares Means 2020. Available from: https://cran.r-project.org/package=emmeans.

[38] Golding M, Doyle K, Sindhusake D, Mitchell P, Newall P, Hartley D. Tympanometric and acoustic stapedius reflex measures in older adults: the Blue Mountains Hearing Study. J Am Acad Audiol. 2007;18(05):391–403. doi: 10.3766/jaaa.18.5.4 17715649

[39] Korczak PA, Kurtzberg D, Stapells DR. Effects of sensorineural hearing loss and personal hearing aids on cortical event-related potential and behavioral measures of speech-sound processing. Ear and hearing. 2005;26(2):165–85. doi: 10.1097/00003446-200504000-00005 15809543

[40] Presacco A, Innes-Brown H, Goupell MJ, Anderson S. Effects of Stimulus Duration on Event-Related Potentials Recorded From Cochlear-Implant Users. Ear Hear. 2017;38(6):e389–e93. Epub 2017/05/06. doi: 10.1097/AUD.0000000000000444 ; PubMed Central PMCID: PMC5659925. 28475545 PMC5659925

[41] Pratt S, Lightfoot G. Physiological mechanisms underlying MLRs and cortical EPs. Translational perspectives in auditory neuroscience: Hearing across the life span-assessment and disorders. 2012.

[42] Kosaner J, Van Dun B, Yigit O, Gultekin M, Bayguzina S. Clinically recorded cortical auditory evoked potentials from paediatric cochlear implant users fitted with electrically elicited stapedius reflex thresholds. International journal of pediatric otorhinolaryngology. 2018;108:100–12. doi: 10.1016/j.ijporl.2018.02.033 . 29605337

[43] Távora-Vieira D, Mandruzzato G, Polak M, Truong B, Stutley A. Comparative Analysis of Cortical Auditory Evoked Potential in Cochlear Implant Users. Ear and hearing. 2021;42(6):1755–69. doi: 10.1097/AUD.0000000000001075 34172688

